# A Lot Can Happen in a Decade

**DOI:** 10.1371/journal.pbio.1001689

**Published:** 2013-10-22

**Authors:** Emma Ganley

**Affiliations:** PLOS Biology, Public Library of Science, Cambridge, United Kingdom

## Abstract

*PLOS Biology* turns ten and, as part of our Tenth Anniversary Collection, Senior Editor Emma Ganley notes how the publishing landscape has changed over the past decade.

This issue marks the 10-year anniversary of *PLOS Biology*, and it's as good a time as any to pause and take stock of how the last decade, PLOS, and *PLOS Biology* have seen irreversible changes in academic publishing. And these changes are for the better.

Historically, individual decades have seen changes in social norms and attitudes, changes that have, very occasionally, resulted in the unthinkable becoming…well, thinkable. This year, for example, the United States marked the 50^th^ anniversary of Martin Luther King's ‘I have a dream’ speech – a highlight of the American civil rights movement that achieved many important goals during a single decade and was in turn celebrated in the first decade of the 21^st^ century by the election of the first African American to the office of President of the United States. Political and social change on this kind of scale requires not only attitudinal change—often brought about by campaigning communities—but also legislative support to act as the foundation for sustained change. For the Open Access movement, the last 10 years have been a pivotal time for addressing the financial and commercial considerations of academic publishing, moving from grass roots initiatives to the introduction of government policy changes.

Over this same 10-year span of time, the world as we know it witnessed an acceleration of technological transformation, driven in large part by advanced internet and new communication technologies, which have easily kept pace with exponential growth predictions made from Moore's law [1]. In the developed world, smartphones and tablets abound, and the world is connected for more hours per day than is probably healthy. And in developing countries, some non-profits, like the One Laptop per Child initiative [2], have sought to ensure that the younger generation in these countries won't be left in the technological dark ages.

From year to year, scientific, technical, and medical (STM) research has also advanced in leaps and bounds, with corresponding increases in the number of research publications (PubMed lists 1,059,583 papers published in 2012 compared with 561,605 in 2002). Studies in the humanities and social sciences also forge on with new findings and theories being published all the time. With the benefit of technology, the results of these academic pursuits can be published online and that one online copy is, theoretically, within the reach of everyone, everywhere. But is this really the case? Over the last decade, there has been an immense effort to change how accessible all of this new (and old) information is to the world at large. At *PLOS Biology*, we all feel immense pride in our 10-year involvement in the Open Access movement, which has seen a wide uptake and acceptance of open access publishing. Further progress toward greater accessibility occurred earlier this year with the introduction of a bill to the United States Senate and House of Representatives—the Fair Access to Science and Technology Research Act [3] (successor to the Federal Research Public Access Act)—which would mandate earlier public release of taxpayer-funded research. In the United Kingdom, the 2012 Finch report recommended that the United Kingdom support ‘gold’ open access publishing for immediate access to papers upon publication [4]. The response by Research Councils UK took the form of a new policy on open access, effective in 2013, to provide grants to UK Higher Education Institutes to support payment of article processing charges associated with open access publishing. The European Commission has a strategy in place that aims to make the results of projects funded by the EU Research Framework open access via either ‘green’ or ‘gold’ publishing. Australian Research Councils (ARC) implemented a policy at the start of 2013 requiring deposition of ARC-funded research publications in an open access institutional repository within 12 months of publication. Discussions about the costs during this transition phase are ongoing, and there remains a question of whether pushing for ‘green’ or ‘gold’ is the better route to take. But, regardless of its ‘colour’, the future for improved access to research is definitely bright.

Although PLOS wasn't the first open access publisher (BioMed Central, the first large-scale publisher devoted to open access, launched in 2000 and the Hindawi Publishing Corporation was also already on the scene), we have, we feel, played a pivotal role alongside these and other kindred publishers in promoting and supporting the progression and uptake of the Open Access movement. In a study published in 2011 in *PLOS ONE*, Laasko et al. discussed how open access publishing developed between 1993 and 2009, reporting an estimated 191,000 articles published in 2009 in 4,769 open access journals; an average annual growth rate of 18% in the number of open access journals from the year 2000, and an average annual growth rate of 30% in the number of open access articles. At the time of this writing, the Directory of Open Access Journals (also coincidentally celebrating their tenth anniversary this year—Happy Anniversary, DOAJ!) contains 9,901 journals and 1,503,096 articles. What an amazing achievement.

After many scientists did not follow through on their initial pledges to support open access publishing, PLOS' founders saw the need to fill a publishing hole, their intention being to create more open access venues where scientists could publish. The launch of PLOS had the additional desired effect of creating more pressure on traditional publishers to consider their business models. And, too, it met the need to dispel a myth— demonstrating that open access publishing did not equal vanity publishing, even though it is the author who pays the costs associated with publishing in this model. PLOS also showed that open access publishing could be done in a way that might tempt scientists to submit their best work to somewhere other than the established traditional journals.

In the last decade, PLOS has grown from publishing just two flagship journals (*PLOS Biology*, closely followed by *PLOS Medicine*), to quickly adding a suite of Community Journals, to finally, in 2007, launching *PLOS ONE*. And while PLOS has stopped there, the relatively small number of journals has not limited our capacity to publish large numbers of articles; on the day of this writing, *PLOS ONE* had 72,774 papers in PubMed. This demonstrated success of high-volume publishing has been instrumental in the launch of several other similarly modelled open access journals by other publishers. In more recent times, another fundamental shift in those providing implicit support for the Open Access movement occurred when three international research funders – The Wellcome Trust, the Max Planck Society, and the Howard Hughes Medical Institute – confirmed their commitment to open access with the launch of their own journal, *eLife* [6].

But has the progress in the last decade achieved everything that we might have wanted for open access publishing? One of *PLOS Biology*'s long time editorial board members, Tom Misteli, noted to us recently that, ‘*PLOS Bio[logy]* has been a catalyst for change in the publishing landscape and… has changed the way we think about publishing.’ Success has definitely been achieved, but arguably, the focus is now altered. How can we lure those authors who routinely send papers to those subjectively perceived ‘top-tier’ closed access journals so they might, instead, submit their papers for publication in an open access journal? One key area that needs a lot of work is to redress how academic and other institutions assess research output for career progression. It seems inappropriate that researchers' work, and indeed the researchers themselves, are ranked according to the impact factor of the journals in which the work is published, a metric derived from non-open, unavailable data [7],[8]. Much better to assess the overall merits and the perceived value of the research by making use of all of the article-level metrics that are readily available from various sources about a piece of published work [9].

Another important area to consider is whether all forms of open access publishing are equal. One key purpose of providing access is to enable and facilitate reuse of the content, but the licenses publishers use can vary radically from one journal to the next. If a paper is ‘open’ via deposition in a repository, or as part of a publisher's hybrid access model, it may still, unfortunately, remain ‘closed’ from a reuse perspective. In an effort to detail the intricacies of the different licenses and what they all mean, PLOS put together an Open Access Spectrum. We recommend you take a look: http://www.plos.org/about/open-access/howopenisit/.

Taking the extension of open access a step further, all of us at *PLOS Biology* are very keen to push availability of and access to data as well as to the article itself. Very commonly, the data on which research articles draw are not freely available alongside the published article. There are some longstanding databases for easily housed data types, and we've seen some new initiatives in the past few years that have facilitated deposition and ‘publication’ of corresponding data (Figshare, Dryad, JCB DataViewer [10]). At *PLOS Biology*, we have an established link with Dryad to facilitate the deposition of datasets alongside your publication [11]. But there is a long way yet to go, with issues around who should be responsible for maintaining data, how that maintenance is funded, whether it is required by funders and publishers, and, not least, making sure to establish good lab practices to prevent loss of data and to enable sharing upon publication. Moving the community's standards and norms for data archiving and sharing to a more consistent and acceptable level will probably need more input from the scientists themselves and their institutes, and may well require ‘incentivizing’ from funders to make sure that open access to data stays on a par with open access publishing. Nevertheless, PLOS is working on new policies for data standards.

In sum, the open access mission is well underway, but it has not yet been fully accomplished. At PLOS, we are delighted to welcome Elizabeth Marincola, our new Chief Executive Officer, who brings with her such wealth of experience that we're sure that PLOS is about to embark on a new era. And as we enter our second decade, we believe *PLOS Biology*, like PLOS itself, has an expanded mission to improve all aspects of scientific communication. Open access was just the first step. Editorial board member Ann Stock summed up nicely our importance with her view that, ‘*PLOS Biology* provides to the publishing community what its articles provide to the scientific community, a force that pushes the envelope into previously unexplored areas’. All of us at *PLOS Biology* are excited by the challenges that remain. We look forward to experimenting further and continuing our endeavours to change the publishing landscape and to making sure that STM publishing evolves to keeps pace with the scientific and technological times.

And last, but by no means least, we want to thank our editors, reviewers, authors, and readers for their support over the last decade – given generously to what was initially a fledgling journal that managed to make quite a big splash in the vast STM publishing pool. Our editors, especially, work with us tirelessly to maintain our unique partnership of professional and academic editorial oversight for all papers that we review and publish. The support we've received has continued and grown over the last decade, making *PLOS Biology* a high-ranking, leading open access journal. We are grateful for every moment you spend working with us to assess and/or review manuscripts along the way. We have a loud voice, we like being controversial, and we urge you to continue working with us to help us innovate, keep on pushing boundaries, and make big ripples on the publishing pool. We love making your fascinating science open to the world, so please carry on sending us your best data and manuscripts!
